# Combining Robotic Navigational Bronchoscopy and Lung Resection Into a Single Anesthetic Event: Cost-Effectiveness, Wait Times, and Outcomes

**DOI:** 10.1016/j.atssr.2024.11.008

**Published:** 2024-12-05

**Authors:** Lucas Weiser, Claire Perez, Justin J. Watson, Kellie Knabe, Allen Razavi, Vikram Krishna, Charles Fuller, Sevannah Soukiasian, Andrew R. Brownlee, Harmik J. Soukiasian

**Affiliations:** 1 Division of Thoracic Surgery, Department of Surgery, Cedars-Sinai Medical Center, Los Angeles, California

## Abstract

**Background:**

Delay from diagnosis to resection in non-small cell lung cancer negatively affects survival. Single-anesthesia robotic navigational bronchoscopy with biopsy and lung resection (SABR) was developed to mitigate delay. We report outcomes, wait times, and cost effectiveness of this approach vs staggered robotic navigational bronchoscopy followed by resection.

**Methods:**

Patients undergoing SABR or staggered biopsy and resection between April 2020 and May 2023 were included. Demographic and procedural characteristics were collected for both groups. Time from clinic and biopsy to operation, length of stay, and inpatient complications were captured. Direct, indirect, and total costs, adjusted to 2023 dollars, were measured for each case.

**Results:**

The study included 40 patients in the SABR group and 30 patients in the staggered group. Baseline clinical characteristics including pathologic stage were similar between the groups. No difference was found in rates of complications. Time from clinic to resection was 26.5 days in the SABR group and 41.5 days in the staggered group (*P* = .001). Time from biopsy to resection was 0 days for the SABR group, whereas it was 17 days for the staggered group (*P* < .001). The SABR group had lower adjusted direct cost ($37,154 vs $41,678; *P* = .005), indirect cost ($33,450 vs $39,706; *P* = .004), and total cost ($70,591 vs $81,025; *P* = .004).

**Conclusions:**

Our experience suggests that the SABR technique offers a significant reduction in the time from diagnosis to treatment as well as reduces cost of care for patients with early-stage non-small cell lung cancer.

**In Short**

▪ The single-anesthetic bronchoscopy and resection technique (SABR) has similar immediate postoperative outcomes to a staggered approach.
▪ SABR significantly decreases delays from clinic to operation and is a cost-effective technique for diagnosis and treatment of early-stage lung cancer.

Lung cancer is the most common cause of cancer-related death in men and women throughout the United States and worldwide.^1^ Anatomic lung resection is the standard of care for early-stage non-small cell lung cancer, and pathways from diagnosis to treatment are highly variable.^2^ Delays between diagnosis and treatment have been shown to result in increased overall risk and decreased survival.^3^, ^4^, ^5^

Increased lung cancer screening and incidental nodule detection have resulted in an increased need for accurate diagnosis of early-stage lesions.^6^ Robotic navigational bronchoscopy (RNB) with biopsy is a promising new technology that has shown superior diagnostic yields with low complication rates compared with traditional bronchoscopic biopsy.^7^ As such, it is becoming a viable diagnostic modality for surgeons and patients.

To mitigate delays in treatment as diagnostic capabilities improve, a single-anesthetic bronchoscopy and resection (SABR) technique has been developed. This technique allows real-time diagnosis by RNB with biopsy, followed by immediate formal oncologic resection as indicated. With the high diagnostic yield of RNB, the SABR technique has been demonstrated to be a safe and effective treatment in select cases.^8^ The aim of this study was to provide insight into differences in overall cost, delays in treatment, and outcomes in SABR compared with a traditional staggered biopsy followed by anatomic resection.

## Material and Methods

In this retrospective analysis, patients undergoing combined or staggered biopsy and anatomic lung resection between April 2020 and May 2023 were included for analysis. Patients were excluded if they underwent induction therapy, did not undergo an anatomic resection, or had resection for benign disease; 30 patients underwent the staggered approach and 40 patients underwent a combined technique, or SABR, and were included in analysis. This study was approved by an institutional review board.

Baseline demographic information was obtained for each patient. Tumor characteristics were captured for both the SABR and staggered groups and included extent of operation, size of tumor, and stage. Extent of operation included segmentectomy and lobectomy. Size of tumor was determined by documented radiographic size.

The primary outcome measure was cost associated with both the SABR and staggered groups. Cost data were obtained from a hospital cost accounting database and included supplies, operative time, postoperative care, and readmission. Direct, indirect, and total costs, all adjusted to 2023 dollars, were measured and compared. Direct costs were extracted from the hospital cost accounting database and included time, supplies, and other expenses directly related to patient care. Indirect costs included those not directly associated with patient care, such as overhead for education and research. Secondary outcomes included overall outcome and delays from diagnosis to operation. Overall outcome is represented by inpatient length of stay and diagnosis of inpatient complications.

Statistical tests included Student *t*-test, Fisher exact test, and Pearson *χ*^2^ test as indicated. Statistical significance was determined as a *P* value < .05. All statistical analysis was performed in R (R Foundation for Statistical Computing).

## Results

A total of 566 patients underwent RNB, with 104 requiring surgical resection; 70 met inclusion criteria, with 40 patients in the SABR group and 30 patients in the staggered group. Groups were similar overall, with no statistically significant differences identified (Table 1). The median age was 69 (interquartile range [IQR], 65.5-78) years and 75 (IQR 77-96) years for the SABR and staggered groups, respectively (*P* = .24). In the SABR group, most patients were female, whereas the staggered group had an even distribution of male and female patients (*P* = .14). There were no major differences in demographics or diagnosed comorbid conditions in either group. Median forced expiratory volume in 1 second was 100% (IQR, 85%-119%) and diffusing capacity of the lung for carbon monoxide was 83% (IQR, 77%-96%) for the SABR group and 96% (84%-116%) and 73.5% (61%-92%) for the staggered group (*P* = .71; *P* = .12).

**Table 1:** Demographic Information for Patient Cohorts

Variable	SABR	Staggered	*P* Value
	(n = 40)	(n = 30)	
Age, y	69 (65.5-78)	75 (77-96)	.24
Sex			.14
Male	13 (32.5)	15 (50.0)	
Female	27 (67.5)	15 (50.0)	
Race			.14
Asian	5 (12.5)	0 (0.0)	
Black	5 (12.5)	2 (6.67)	
White	29 (72.5)	27 (90.0)	
Other	1 (2.50)	1 (3.33)	
Smoking status			0.62
Current	4 (10.0)	5 (16.7)	
Former	28 (70.0)	18 (60.0)	
Never	8 (20.0)	7 (23.3)	
Medical history			
Diabetes	5 (12.5)	3 (10.0)	.28
COPD	5 (12.5)	4 (13.3)	.99
CAD	5 (12.5)	4 (13.3)	.99
Renal disease	0 (0.0)	3 (10.0)	.07
Prior biopsy	2 (5.00)	6 (20.0)	.07
Prior cancer	15 (37.5)	10 (33.3)	.72
Pulmonary function			
% FEV_1_	100 (85.0-119)	96 (84.0-116)	.71
% Dlco	83.0 (77.0-96.0)	73.5 (61.0-92.0)	.12

Categorical variables are presented as number (percentage). Continuous variables are presented as median (lower quartile–upper quartile).

CAD, coronary artery disease; COPD, chronic obstructive pulmonary disease; Dlco, diffusing capacity of the lung for carbon monoxide; FEV_1_, forced expiratory volume in 1 second; SABR, single-anesthetic bronchoscopy and resection.

Tumor characteristics were similar between groups (Table 2). Most patients underwent lobectomy, with 35 (87.5%) patients in the SABR group and 26 (86.7%) patients in the staggered group (*P* = .99). Median tumor size was 2.15 cm (IQR, 1.5-2.55 cm) and 2.10 cm (IQR, 1.5-2.55 cm) in the SABR and staggered groups, respectively (*P* = .29). Most patients had stage I disease in both groups, and 4 cases in the SABR group were designated metastasectomies (*P* = .22).

**Table 2:** Tumor Characteristics of Patient Cohorts

Variable	SABR	Staggered	*P* Value
	(n = 40)	(n = 30)	
Extent of operation			.99
Lobectomy	35 (87.5)	26 (86.7)	
Segmentectomy	5 (12.5)	4 (13.3)	
Tumor size			.29
Radiographic size, cm	2.15 (1.50-2.55)	2.10 (1.50-2.55)	
Pathologic stage			.22
I	27 (67.5)	23 (76.7)	
II	5 (12.5)	4 (13.3)	
III	4 (10.0)	3 (10.0)	
Metastasis	4 (10.0)	0 (0.0)	

Categorical variables are presented as number (percentage). Continuous variables are presented as median (lower quartile–upper quartile).

SABR, single-anesthetic bronchoscopy and resection.

Median days from clinic to operation were 26.5 (IQR, 19-34) days in the SABR group compared with 41.5 (IQR, 29-61) days in the staggered group (*P* = .001). Time from biopsy to resection was lower for SABR patients, 0 (IQR, 0-0) days compared with 17 (IQR, 7-39) days in the staggered group (*P* < .001). Length of stay was similar between groups (*P* = .96). Air leak, pneumonia, atrial fibrillation, and overall complication rates were similar between groups (Table 3).

**Table 3:** Comparison of Outcomes and Cost Between Groups

Variable	SABR	Staggered	*P* Value
	(n = 40)	(n = 30)	
Time factors, d			
Clinic to operation	26.5 (19.0-34.0)	41.5 (29.0-61.0)	**.001**
Biopsy to resection	0 (0-0)	17.0 (7.00-39.0)	**<.0001**
Length of stay	3.50 (2.50-4.00)	3.00 (2.00-5.00)	.96
Complications			
Air leak	3 (7.50)	2 (6.67)	.99
Pneumonia	1 (2.50)	3 (10.0)	.31
Atrial fibrillation	3 (7.50)	2 (6.67)	.99
Overall complication rate	9 (22.5)	9 (30.0)	.48
Adjusted cost (per case)			
Direct	$37,154 (30,934-41,023)	$41,678 (36,151-50,097)	**.005**
Indirect	$33,450 (30,072-37,460)	$39,706 (33,444-43,817)	**.004**
Total	$70,591 (62,515-77,080)	$81,025 (67,136-91,307)	**.004**

Categorical variables are presented as number (percentage). Continuous variables are presented as median (lower quartile–upper quartile). Boldface *P* values represent statistical significance.

SABR, single-anesthetic bronchoscopy and resection.

Overall cost per case was lower in the SABR group than in the staggered group (Table 3). The median direct cost per case was $37,154 (IQR $30,934-$41,023) for the SABR group and $41,678 (IQR $36,151-$50,097) for the staggered group (*P* = .005). Indirect cost showed a similar trend, with the SABR and staggered group having a median cost of $33,450 ($30,072-$37,460) and $39,706 ($33,444-$43,817), respectively (*P* = .004). Median total cost was $70,591($62,515-$77,080) and $81,025 ($67,136-$91,307) for the SABR and staggered groups, respectively (*P* = .002). The overall difference in cost between the SABR and staggered groups, as defined by the difference between the median values, was $10,434 per case (Figure).

**Figure:**
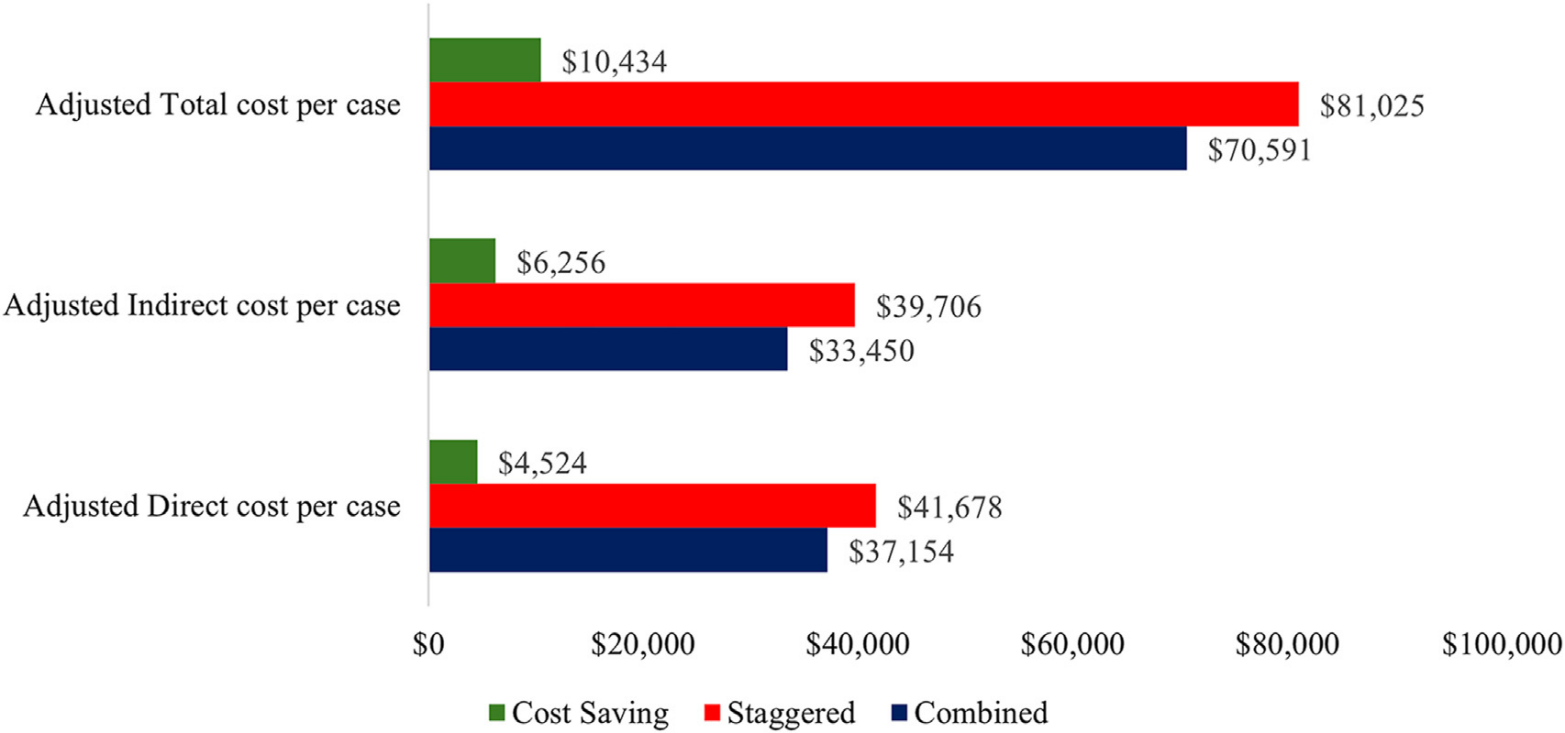
Cost savings per case with single-anesthetic bronchoscopy and resection technique.

## Comment

This investigation demonstrates that SABR has similar outcomes to a traditional staggered method while being less expensive and significantly reducing delays from clinic and diagnosis to operation. The difference in time from clinic to operation between groups is likely to be accounted for by the difference in time from biopsy to operation. The SABR group, by definition, had no time difference between diagnosis and operation, directly affecting the time from clinic to resection. As such, SABR results in a 36.1% decrease in time from clinic to operation compared with the traditional method. With more and more evidence demonstrating that delays in operation have an impact on overall survival, options to decrease delays should be considered in developing a treatment plan for suspected pulmonary malignant disease.

Postoperative outcomes were statistically similar between groups; however, they represent only a small number of metrics that can be used to understand overall outcome after an oncologic procedure. Further study is warranted to evaluate unplanned hospital readmission as well as short- and long-term cancer-free survival. Given that patients in both groups had the same definitive management and that there was no statistically significant difference between the extent of operation, it is unlikely that there will be longer term differences in outcome.

In this study, patients were excluded if they did not have a clear diagnosis on biopsy. The traditional method with staggered biopsy and resection involves full pathologic review of the biopsy specimen with final diagnosis. The combined method, however, involves a frozen pathologic specimen and an expedited diagnosis. Studies have shown that the diagnostic yield of the robotic navigation biopsy is 87.9%, with increase in yield associated with nodule size greater than 2.1 cm,^8^ significantly higher than in typical bronchoscopic biopsy.^9^^,^^10^ Frozen pathology, however, is dependent on multiple variables beyond the adequacy of the biopsy specimen. It is imperative that this be considered in performing a combined approach to minimize the risk of a false-negative result.

Median indirect, direct, and total costs per case for the SABR group were significantly lower than for the staggered group. The likely cause of this finding is the requirement of 1 visit to the preoperative area, 1 induction of anesthesia, and 1 visit to the postoperative area. Time in the operating room is expensive, and although the overall operative time is likely to be longer when biopsy must be performed before resection, eliminating a second visit to the hospital and operating room seems to outweigh this cost. With a difference in median total cost of $10,434 per case, this SABR cohort saved approximately $417,360. Ongoing improvement in the technique to decrease operative time is likely to further its cost-effectiveness. This study was conducted at a tertiary care facility with a robust and thorough billing and accounting department. As such, cost includes advanced amortization of instruments and devices and may be higher than the reported national average in general.

As a single-institution study, the diagnostic yield of the frozen biopsy as well as the cost analysis is not necessarily generally applicable. Being a new technique, the overall number of patients in each cohort was low, which may have had an impact on the statistical power of the study. This analysis also does not investigate short- or long-term oncologic outcomes and reports only immediate postoperative outcomes.

Despite these limitations, this analysis demonstrates that the SABR technique is cost-effective, significantly decreases harmful delays from diagnosis to operation, and has similar immediate postoperative outcomes to a staggered approach at this institution. This approach represents a viable new option in expediting surgical management of early-stage lung cancer patients. Further study, particularly larger, multi-institutional investigations, are warranted to better understand the patient- and cost-related benefits of the SABR technique.
